# 25(OH)D Levels in Infancy Is Associated With Celiac Disease Autoimmunity in At-Risk Children: A Case–Control Study

**DOI:** 10.3389/fnut.2021.720041

**Published:** 2021-08-11

**Authors:** Carin Andrén Aronsson, Xiang Liu, Jill M. Norris, Ulla Uusitalo, Martha D. Butterworth, Sibylle Koletzko, Suvi M. Virtanen, Iris Erlund, Kalle Kurppa, William A. Hagopian, Marian J. Rewers, Jin-Xiong She, Jorma Toppari, Anette-G. Ziegler, Beena Akolkar, Jeffrey P. Krischer, Daniel Agardh

**Affiliations:** ^1^Department of Clinical Sciences, Lund University, Malmö, Sweden; ^2^Department of Pediatrics, Health Informatics Institute, Morsani College of Medicine, University of South Florida, Tampa, FL, United States; ^3^Department of Epidemiology, Colorado School of Public Health, University of Colorado Denver, Aurora, CO, United States; ^4^Dr. von Hauner Children's Hospital, Ludwig Maximilians University, Munich, Germany; ^5^Department of Pediatrics, Gastroenterology and Nutrition, School of Medicine Collegium, Medicum University of Warmia and Mazury, Olsztyn, Poland; ^6^Department of Public Health Solutions, Finnish Institute for Health and Welfare, Helsinki, Finland; ^7^Unit of Health Sciences, Faculty of Social Sciences, Tampere University, Tampere, Finland; ^8^Center for Child Health Research, Tampere University, Tampere, Finland; ^9^Department of Government Services, Finnish Institute for Health and Welfare, Helsinki, Finland; ^10^Tampere Center for Child Health Research, Tampere University, Tampere, Finland; ^11^Department of Pediatrics, Tampere University Hospital, Tampere, Finland; ^12^The University Consortium of Seinäjoki, Seinäjoki, Finland; ^13^Department of Pediatrics, Seinäjoki Central Hospital, Seinäjoki, Finland; ^14^Pacific Northwest Research Institute, Seattle, WA, United States; ^15^Barbara Davis Center for Childhood Diabetes, University of Colorado, Aurora, CO, United States; ^16^Center for Biotechnology and Genomic Medicine, Medical College of Georgia, Augusta University, Augusta, GA, United States; ^17^Department of Pediatrics, Turku University Hospital, Turku, Finland; ^18^Institute of Biomedicine, Research Centre for Integrative Physiology and Pharmacology, Centre for Population Health Research, University of Turku, Turku, Finland; ^19^Institute of Diabetes Research, Helmholtz Zentrum München, Klinikum rechts der Isar, Technische Universität München, and Forschergruppe Diabetes e.V., Neuherberg, Germany; ^20^National Institute of Diabetes and Digestive and Kidney Diseases, Bethesda, MD, United States

**Keywords:** celiac disease autoimmunity, infants, children, vitamin D, TEDDY, celiac disease

## Abstract

**Objectives:** An observed variation in the risk of celiac disease, according to the season of birth, suggests that vitamin D may affect the development of the disease. The aim of this study was to investigate if vitamin D concentration is associated with the risk of celiac disease autoimmunity (CDA) in genetically at-risk children.

**Study Design:** Children prospectively followed in the multinational The Environmental Determinants of Diabetes in the Young study, conducted at six centers in Europe and the US, were selected for a 1-to-3 nested case–control study. In total, 281 case–control sets were identified. CDA was defined as positivity for tissue transglutaminase autoantibodies (tTGA) on two or more consecutive visits. Vitamin D was measured as 25-hydroxyvitamin D [25(OH)D] concentrations in all plasma samples prior to, and including, the first tTGA positive visit. Conditional logistic regression was used to examine the association between 25(OH)D and risk of CDA.

**Results:** No significant association was seen between 25(OH)D concentrations (per 5 nmol/L increase) and risk for CDA development during early infancy (odds ratio [OR] 0.99, 95% confidence interval [CI] 0.95–1.04) or childhood (OR 1.02, 95% CI 0.97–1.07). When categorizing 25(OH)D concentrations, there was an increased risk of CDA with 25(OH)D concentrations <30 nmol/L (OR 2.23, 95% CI 1.29, 3.84) and >75 nmol/L (OR 2.10, 95% CI 1.28–3.44) in early infancy, as compared with 50–75 nmol/L.

**Conclusion:** This study indicates that 25(OH)D concentrations <30 nmol/L and >75 nmol/L during early infancy were associated with an increased risk of developing CDA in genetically at-risk children. The non-linear relationship raises the need for more studies on the possible role of 25(OH)D in the relation to celiac disease onset.

## Introduction

Vitamin D is essential for bone growth and the effective functioning of innate immunity (1). Most of the circulating vitamin D, 25-hydroxyvitamin D, [25(OH)D] is synthesized by the skin with the help of UVB radiation from sunlight. Populations living in the northern hemisphere often need to compensate for the lower amount of sunlight *via* fortified foods or dietary supplements (2). Interestingly, vitamin D sufficiency, compared with lower than normal plasma levels, was associated with decreased risk of developing autoimmunity or immune-mediated diseases such as type 1 diabetes (T1D) (3–5), multiple sclerosis, rheumatoid arthritis, and Crohn's disease (6–8). Celiac disease is a chronic enteropathy with autoimmune features caused by an immune-mediated response to dietary gluten, leading to the destruction of intestinal mucosa resulting in malabsorption (9). Celiac disease autoimmunity (CDA) is defined as the presence of tissue transglutaminase autoantibodies (tTGA), indicative of an ongoing gluten-induced inflammatory response, which often precedes small bowel mucosal damage and a celiac disease diagnosis (10, 11). Although celiac disease is strongly associated with human leucocyte antigen (HLA) DQA1^*^05:01-DQB1^*^02 and DQA1^*^03:01-DQB1^*^03:02 haplotypes, these risk genes cannot fully explain the risk, suggesting that environmental factors also contribute (9). The most promising promoters so far are gastrointestinal infections (12, 13), high gluten intake (14), or both (15). For reasons yet unknown, children born during the spring and summer months are at the highest disease risk (16–20). This has led to the hypothesis that vitamin D deficiency in early life may predispose to celiac disease due to seasonal differences in UVB exposure and subsequent 25(OH)D concentrations or *via* dysregulation of the immune response leading to an abnormal intestinal mucosa with increasing permeability (21, 22). Although low 25(OH)D concentrations have been reported at the time of celiac disease diagnosis (23–26), this can be attributed to deranged dietary absorption from a damaged gut epithelium.

The aim of this study was to investigate if the levels of 25(OH)D before the disease onset are associated with increased risk of CDA in a prospective birth cohort of genetically at-risk children.

## Materials and Methods

### Study Population

The Environmental Determinants of Diabetes in the Young (TEDDY) is a prospective birth cohort consisting of 8,676 genetically at-risk children born between September 2004 and February 2010. Children were enrolled in the study before the age of 4.5 months and followed for 15 years to identify environmental triggers of T1D and celiac disease (27, 28). Children carrying high-risk HLA alleles for T1D and celiac disease were enrolled at six centers, three in the US (Colorado, Washington, and Georgia/Florida) and three in Europe (Finland, Germany, and Sweden). The following HLA-class II genotypes: HLA-DR3/4; HLA-DR4/4, HLA-DR4/8, HLA-DR3/3, and HLA-DR4/4 were the eligibility criteria for enrollment in the study. Children with HLA-DR4/1, HLA-DR4/13, HLA-DR4/9, and HLA-DR3/9 were included if they had a first degree relative (FDR) i.e. having a mother, father, or sibling with T1D.

For all study participants, separate written informed consent was obtained from a parent or primary caretaker for genetic screening and participation in the prospective follow-up beginning at birth. The study was conducted according to the guidelines of the Declaration of Helsinki, and local institutional or regional ethics review boards in all participating countries approved the study.

### Study Design

The present study was performed by using children included in two nested case–control cohorts that were constructed with a focus on islet autoimmunity (IA) and T1D, and which aimed to study multiple biomarkers that are expensive to measure in a large birth cohort setting. The design and planning of the TEDDY nested case–control biomarker study have been described in detail elsewhere (29). Cases and controls were identified as of May 31, 2012, and were matched by clinical center, sex, and family history of T1D. All available samples meeting the design criteria by that time were processed in the laboratories chosen for each biomarker analysis. All children in the 1:3 nested case–control studies for vitamin D biomarker that had been screened for CDA as of August 31, 2017 were considered for the present study. Each case–control set included a CDA positive child (“case”) matched up to three controls. All controls were tTGA-negative for at least 6 months from the age of seroconversion of the case. Subjects having at least one 25(OH)D measurement prior or at seroconversion visit of the matched cases were included in the final analysis, resulting in 281 cases and 643 controls (123 cases with three matched controls, 116 cases with two controls, and 42 cases with one control) (Supplementary Figure 1). Maternal and early infant feeding information were collected prospectively at clinic visits every 3 months. Descriptive characteristics of the study population are presented in Table 1.

**Table 1:** Descriptive characteristics of subjects in the nested case–control study.

	**Cases (*n* = 281)**	**Controls (*n* = 643)**
**Characteristic**	***N* (%) or median**	***N* (%) or median**
	**(Q1, Q3)**	**(Q1, Q3)**
Female sex (yes)^*^	144 (51.2)	320 (49.8)
Clinical Center^*^		
Finland	64 (22.8)	147 (22.9)
Germany	27 (9.6)	52 (8.1)
Sweden	116 (41.3)	275 (42.8)
Colorado	36 (12.8)	83 (12.9)
Washington	16 (5.7)	43 (6.7)
Georgia	22 (7.8)	43 (6.7)
**HLA genotype**		
DR3/3	105 (37.4)	90 (14.0)
DR3/X	121 (43.1)	261 (40.6)
Other	55 (19.6)	292 (45.4)
Long distance protocol^a^ (yes)	43 (15.3)	108 (16.8)
FDR with type 1 diabetes (yes)^*^	68 (24.2)	152 (23.6)
FDR with celiac disease (yes)	27 (9.6)	26 (4.0)
Age at CDA (years)	2.8 (2.0, 3.9)	NA
Developed celiac disease during follow-up (yes)	102 (36.3)	NA
Persistent confirmed islet autoantibodies (yes)	83 (29.5)	146 (22.7)
Islet autoantibody positivity prior to CDA (yes)	55 (19.6)	NA
**Season of birth**		
Spring (Mar–May)	75 (26.7)	149 (23.2)
Summer (Jun–Aug)	68 (24.2)	171 (26.6)
Fall (Sep–Nov)	61 (21.7)	173 (26.9)
Winter (Dec–Feb)	77 (27.4)	150 (23.3)
**Breastfeeding duration (months)**		
Exclusive	0.9 (0.0, 4.0)	0.5 (0.0, 3.2)
Any	8.3 (5.4, 12.1)	8.0 (3.8, 12.0)
Age at gluten introduction (months)	6.0 (5.1, 6.9)	6.0 (5.1, 6.9)
**Maternal education**		
Basic primary	57 (20.3)	146 (22.9)
Higher education	224 (79.7)	492 (77.1)
Maternal vitamin D supplementation during pregnancy (yes)	181 (64.4)	406 (63.1)

* *Matching variables for the nested case control study.*

a *Long-distance protocol (LDP); that is, at least one sample was collected locally and shipped to a TEDDY clinic for processing.*

*FDR, first degree relative (mother, father, or sibling); CDA, celiac disease autoimmunity; NA, not applicable*.

### Screening for CDA and Celiac Disease

Annual screening for celiac disease started from the age of 2 years using radiobinding assays (RBA) to measure tTGA in serum. Samples were analyzed at the Bristol Laboratory (University of Bristol, UK) for the European sites and the Barbara Davis Center Laboratory (Aurora, Colorado) for the US sites. In the US, the RBA uses anti-IgA agarose to capture IgA-TGA, whereas, in Bristol, a mixture of both anti-IgA agarose and protein A sepharose is used to assess both IgA-tTG and IgG-tTG. Children from the US sites with a tTGA level > 0.05 were deemed antibody positive. To harmonize the protocol, all sera with tTGA levels > 0.01 at the laboratory in Colorado were reassayed at the laboratory in Bristol for confirmation of being tTGA positive. Children with tTGA levels >1.3 unit in Bristol were deemed antibody positive. All earlier samples of children who were tTGA positive at age 24 months were retrospectively analyzed in the Bristol laboratory to determine the age of seroconversion to tTGA positivity.

Celiac disease autoimmunity was defined as being tTGA positive in two consecutive samples, collected 3–6 months apart. A total of 281 children developed CDA at a median age of the first positive tTGA at 2.8 years of age (IQR; Q1: 2.0, Q3: 3.9 years).

### Vitamin D Assessment

Plasma from blood samples was drawn into light-protected tubes (BD Vacutainer®CPT™ cell preparation tubes) and analyzed at the Institute for Health and Welfare, Helsinki, Finland. 25(OH)D concentration was measured using the ARCHITECT 25-OH vitamin D chemiluminescent microparticle immunoassay (3). The laboratory participates in the vitamin D external quality assessment scheme. Plasma samples for 25(OH)D were collected from visits at 3, 6, 9, and 12 months of age and then annually up or until and including the time of seroconversion for CDA-cases. Vitamin D status was categorized according to cut-offs previously used in pediatric populations, which are as follows: <30 nmol/L, 30–50 nmol/L, 50–75 nmol/L, and >75 nmol/L (30, 31).

### Genotyping

Genotyping was performed by the University of Virginia using a custom Illumina Infinium ImmunoChip (Illumina, Inc; CA). The ImmunoChip was designed to genotype immune-mediated disease loci identified by genome wide association studies in 12 autoimmune diseases (including celiac disease and T1D). Genes in the vitamin D pathway may modify the efficiency of 25(OH) concentrations; therefore several single nucleotide polymorphisms (SNPs) associated with vitamin D metabolism, such as GC (rs7041), VDR (rs1544410 [Bsml], rs11568820 [Cdx2], rs7975232 [Apal]), CYP27B1 (rs4646536), CYP24A1 (rs4809959, rs2616277), and RXRA (rs3818740, rs10881582) were selected for the analyses as previously described (3).

## Statistical Analyses

Conditional logistic regression was used to examine the association between 25(OH)D concentrations and the risk of developing CDA. The magnitudes of the associations were described by odds ratios (ORs) with 95% confidence intervals (CIs). Having an FDR with celiac disease, HLA genotype associated with celiac disease and factors associated with 25(OH)D concentrations including season of drawing blood or season of birth, long-distance protocol (LDP; that is, samples collected locally and shipped to a TEDDY clinic for processing), and IA status (3) were adjusted in the models.

Concentrations of 25(OH)D were examined in early infancy by using the first available sample (i.e., early infancy concentrations) and during childhood, defined as average concentrations of all visits prior and including the seroconversion visit of the case (i.e., childhood concentrations). The early infancy and childhood 25(OH)D concentrations were incorporated into the conditional logistic regression models in two ways, namely: (a) The 25(OH)D concentration as a continuous variable; (b) the 25(OH)D concentration was grouped into four categories as <30, 30–50, 50–75, and >75 nmol/L.

When examining the *a priori* proposed 25(OH)D vitamin D gene interactions with the 25(OH)D concentration, each SNP was analyzed individually by treating the number of minor alleles as a continuous variable, and an interaction term between the SNP and 25(OH)D concentrations was included in the model. Ancestry (population structure) was adjusted in the interaction analyses by using the two largest principal components from a principal component analysis (PCA) of the ImmunoChip data in the cohort, using KING software (32).

All analyses were performed using SAS (Version 9.4; SAS Institute, Cary, North Carolina) version 9.4. A two-sided value of *p* < 0.05 was considered statistically significant.

## Results

### Distribution of 25(OH)D Concentrations

The proportion of children classified as being vitamin D deficient (<30 nmol/L) during early infancy or during childhood were 114 (13.7%) and 49 (5.3%) children, respectively. Early infancy and childhood 25(OH)D concentrations of cases and control subjects according to the matching variables in the study cohort are presented in Supplementary Table 1. Differences in 25(OH)D concentrations were most noticeable between countries and clinical centers, where subjects from Germany and Sweden had the highest concentrations in both early infancy and during childhood. No other major differences in 25(OH)D concentrations for sex and having an FDR with T1D at the two exposure points were observed.

### 25(OH)D Concentrations and Risk of CDA

There was no association between 25(OH)D concentrations and risk for CDA development during early infancy (OR 0.99, 95% CI 0.95–1.04 5 per nmol/L increase) or childhood (OR 1.02, 95% CI 0.97–1.07 5 per nmol/L increase), after adjusting for HLA, FDR with celiac disease, season of birth, season of drawing blood, being on LDP, and IA status (Table 2). There was no interaction between 25(OH)D concentrations and a clinical center on the risk of CDA (*P* = 0.30).

**Table 2:** Association between 25(OH)D concentrations categories and celiac disease autoimmunity (CDA) in children in the nested case–control study.

**25(OH)D concentrations**		**Children with CDA and controls**	
		**OR (95% CI)**	***P***
**Per 5 nmol/L increase**			
Early infancy^a^		0.99 (0.95, 1.04)	0.785
Childhood^b^		1.02 (0.97, 1.07)	0.495
**Concentration categories**			
**Early infancy** ^**a**^	***N*** **(%)**		
<30 nmol/L	114 (13.7)	2.23 (1.29, 3.84)	0.004
30–50 nmol/L	284 (34.0)	1.52 (1.01, 2.28)	0.047
50–75 nmol/L	307 (36.8)	Reference	
>75 nmol/L	130 (15.6)	2.10 (1.28, 3.44)	0.003
**Childhood** ^**b**^			
<30 nmol/L	49 (5.3)	1.38 (0.66, 2.86)	0.39
30–50 nmol/L	313 (33.9)	1.32 (0.90, 1.93)	0.16
50–75 nmol/L	441 (47.7)	Reference	
>75 nmol/L	121 (13.0)	1.30 (0.80, 2.13)	0.29

a *Early infancy is defined as 25(OH)D concentrations at the first available sample at visit up to 12 months of age. Analyses are adjusted for HLA-genotype, season of blood draw, FDR with celiac disease, islet autoantibody status, and long-distance protocol. The first available 25(OH)D sample was at 3 months clinic visit in 66%, 6 months in 17%, 9 months in 10%, and 12 months in 7% of the children.*

b *Childhood is defined as average concentrations of all visits prior and including the (matched cases') seroconversion visit. Analyses are adjusted for HLA-genotype, season of birth, FDR with celiac disease, islet autoantibody status, and long-distance protocol*.

**Figure 1:**
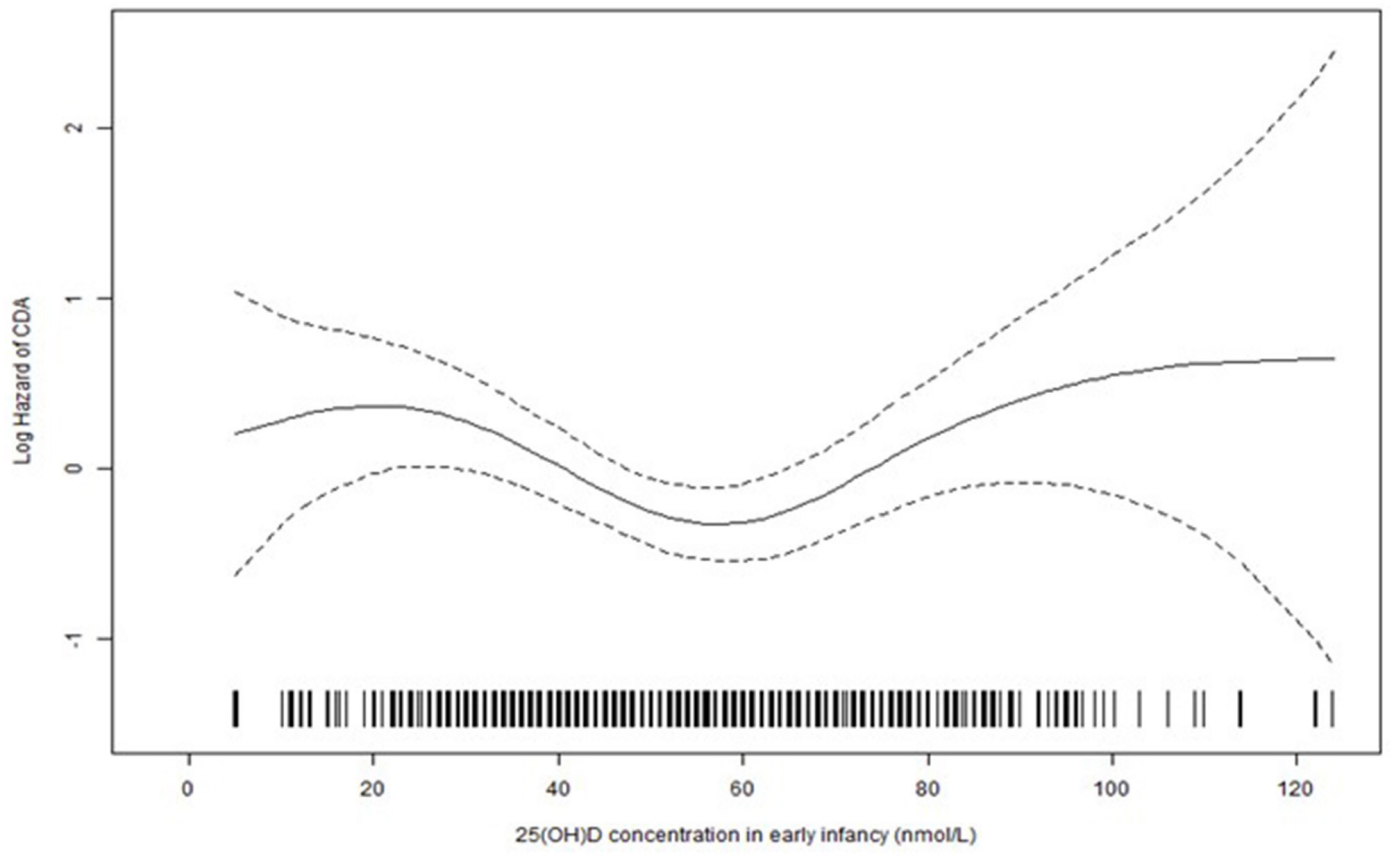
The estimated effect [solid line, with pointwise standard error (dashed lines)] of 25(OH)D concentration in early infancy on the log hazard of CDA from conditional logistic regression analysis with smoothing splines (*p* = 0.014 for non-linearity).

When classifying 25(OH)D concentrations into four categories, there was an increased risk of CDA with 25(OH)D concentrations <30 nmol/L (OR 2.23, 95% CI 1.29, 3.84) and >75 nmol/L (OR 2.10, 95% CI 1.28–3.44) in early infancy compared with 25(OH)D concentrations between 50 and 75 nmol/L (Table 2). No significant association was identified for average childhood 25(OH)D concentrations (Table 2). Using conditional logistic regression analysis with smoothing splines (33, 34), a non-linear relationship between vitamin D concentrations in early infancy and the risk of CDA was observed (*p* = 0.014 for nonlinearity) (Figure 1). The non-linear relationship is consistent with the finding that both <30 nmol/L and >75 nmol/L concentrations in early infancy were associated with a higher risk of CDA compared with 50–75 nmol/L (i.e., a U-shape relationship). Similar non-linear relationships were observed when analyzing the first available sample at 3, 6, and 9 months, respectively (data not shown). In a sensitivity analysis, additional adjustment for gluten consumption prior to seroconversion (14) and being rotavirus vaccinated (12) changed the association for 25(OH)D in early infancy and CDA marginally. Gastrointestinal episodes (12) were not taken into consideration since they could be a mediator rather than a confounder (low vitamin D was associated with higher odds of gastrointestinal infections, *p* = 0.017).

There was no interaction between any of the selected vitamin D pathway SNPs and 25(OH)D concentration, analyzed as both continuous and categorical variables, on CDA (Table 3).

**Table 3:** Test for interaction between vitamin D gene SNPs and 25(OH)D concentrations on the risk of celiac disease autoimmunity in the TEDDY nested case–control study.

		***P*** **-value for SNP interaction** ^**#**^ **with**			
**Gene**	**SNP**	**Early infancy** ^**a**^ **25(OH)D**		**Childhood** ^**b**^ **25(OH)D**	
		**Concentration**	**Concentration categories**	**Concentration**	**Concentration categories**
GC	rs7041	0.49	0.58	0.20	0.52
VDR (Bsml)	rs1544410	0.70	0.56	0.45	0.41
VDR (Cdx2)	rs11568820	0.16	0.13	0.78	0.23
VDR (Apal)	rs7975232	0.56	0.50	0.39	0.21
CYP27B1	rs4646536	0.53	0.67	0.47	0.66
CYP24A1	rs4809959	0.71	0.05	0.54	0.54
CYP24A1	rs2616277	0.13	0.20	0.43	0.78
RXRA	rs3818740	0.27	0.77	0.21	0.34
RXRA	rs10881582	0.88	0.97	0.97	0.26

# *Analyses adjusted for HLA-genotype, the first two PCs indicating ancestry, season of sample collection or season of birth (for childhood 25(OH)D concentration), FDR with celiac disease, long-distance protocol, and islet autoantibody status.*

a *Early infancy is defined as 25(OH)D concentrations at the first available sample at visit up to 12 months of age.*

b *Childhood is defined as average concentrations of all visits prior and including the (matched cases') seroconversion visit*.

To identify potential sociodemographic or other confounding factors explaining the findings, descriptive characteristics of the four 25(OH)D concentration categories are presented in Supplementary Table 2. Among subjects with 25(OH)D concentrations <30 nmol/L, a higher portion of the children were from the sites in Colorado (USA) and Finland, were persistent IA-positive, exclusively breastfed, born during the fall season (September–November), and the blood was drawn during the winter months (December–February). Subjects with 25(OH)D concentrations >75 nmol/L, a higher proportion were from Sweden, reported vitamin D supplementation at the time of drawing the blood, or were on an LDP. Also, only a few subjects were exclusively breastfed and blood drawn was collected during the winter months (December to February).

## Discussion

This nested case–control study found an association of low levels of 25(OH)D concentrations in early infancy with an increased risk of CDA in genetically at-risk children. This finding may shed light on the role of vitamin D in the development of celiac disease. The exact mechanism for this association is not clear, but it could be attributed to the active form of vitamin D (1,25-dihydroxyvitamin D) which inhibits the production of proinflammatory cytokines, possibly leading to a shift from an anti-inflammatory state to a more inflammatory state (35). This speculation would be in line with a previous finding of significantly higher levels for IL-6, IL-13, IL-10, IL-1b, and TNF-α in combination with significantly lower 25(OH)D concentrations in screening-detected celiac disease cases compared with healthy controls (24). These differences were no longer present when comparing celiac disease patients on a gluten-free diet compared with controls. A Norwegian study compared 25(OH)D cord plasma levels between children who later developed celiac disease and healthy controls, and found that levels were highly correlated with maternal 25(OH)D at the time of delivery but not related to later development of celiac disease (36). Drawing conclusions on this is difficult as the 25(OH)D concentrations of the infant at birth are highly dependent on maternal vitamin D status. Early life exposures such as vitamin D supplementation may influence the developmental imprinting of the immune system. Vitamin D is able to modulate the innate immune system and enable the system to fight against pathogens, but it may be so that the infants acquired 25(OH)D status during the first year of life may be more important for shaping the adaptive immune system and risk for later autoimmunity (1, 37).

It has been speculated that earlier identified non-linear relationships between 25(OH)D and different disease outcomes may be related to SNPs and their influence on vitamin D status (38, 39). Most of the circulating 25(OH)D is bound to vitamin D binding protein genes. Identified genes directly involved in the vitamin D metabolism and associated with abnormal 25(OH)D concentrations are VDR, GC, CYP2R1, CYP24A1, DHCR7, and RXRA (40). The functions of these genes can be expressed as a lower affinity for 25(OH)D or impaired 25-hydoxylase activity. In the present study, we included the most critical SNPs involved in the vitamin D pathway as previously described (3), but found no interaction between any of the selected vitamin D pathway SNPs and 25(OH)D, analyzed as both continuous and categorical variables. This is in line with findings in a previous study on neonatal vitamin D status in relation to the later development of celiac disease (36).

Many studies have reported an association between the risk of celiac disease and the season of birth (primarily summer births) (17–19, 41). One possible explanation for this association is the difference in received sunlight in pregnant mothers and spring-born infants, leading to lower 25(OH)D concentrations during the second half of infancy (during fall and winter). The lower vitamin D status in these infants may also coincide with a time when there are frequent seasonal infections and the age when gluten is introduced (~6 months after birth). An alternate hypothesis is that the observed seasonal variation is due to the differences in viral infections, but it may also be that low levels of 25(OH)D increase the susceptibility to infections affecting the gut, causing a disruptive barrier to triggering antigens involved in the celiac disease (22, 42).

A more inexplicable finding from the present study was that 25(OH)D concentrations >75 nmol/L during early infancy increased the risk of CDA in childhood. Concentrations exceeding 75 nmol/L are most likely due to frequent vitamin D supplementation. Interestingly, one previous study reported children who received vitamin D supplementation for longer than 3 months to be at an increased risk of developing celiac disease (43). The proposed explanation is that high doses of vitamin D upregulate Th2 cell cytokines associated with immune reaction to external stimuli (44). In the present study, 92% of the children exceeding 25(OH)D concentrations >75 nmol/L reported vitamin D supplementation at the time of drawing the blood compared with 55% among children with low 25(OHD) concentrations. Differences in 25(OH)D concentrations may also be due to strength, frequency, and the duration of vitamin D supplementation. At the time of the study enrollment, there were differences among countries regarding recommendations for vitamin D supplementation to infants (45). In Sweden and Germany, the vitamin D recommendations (10 μg/day, starting within 2 months of birth) did not change during the study period. In Finland and the US, the recommendations changed from 5 to 10 μg during the corresponding period. Results from the full study cohort showed that nearly 80% of the participants (European sites; 97–99%) started vitamin D supplementation within the 1st year of life (45). Of note, the start of vitamin D supplementation may be a confounder in our analyses because information about the length of supplementation before the time of blood draw was not available. Vitamin D supplementation in higher doses may also be a result of parents knowing the genetic risk of their infants for T1D and supplementing their child as an action to prevent disease. However, in TEDDY, less than 3% of the mothers reported giving dietary supplements as an action to prevent T1D before the age of 2 (46).

At present, there is no clear consensus regarding optimal 25(OH)D concentrations in the general pediatric population. Defining appropriate levels of 25(OH)D has been problematic, and recommendations are mostly based on studies on fracture risk and poorly accounted for the risk of immune-mediated diseases in children.

This study has some limitations. First, the study was originally designed to investigate several dietary biomarkers and its association with IA and T1D status. However, the number of IA-positive subjects was well-balanced between cases (29%) and controls (23%) and adjusted for in the analyses. Another limitation is that a major proportion of 25(OH)D samples close to seroconversion, defined as within 1 year before seroconversion to tTGA positivity, were missing. Samples drawn before seroconversion were only available in <50% of the subjects, resulting in lower power in the statistical tests.

It is debated whether circulating total 25(OH)D is the best marker of vitamin D status compared with the biological active form of vitamin D [1.25(OH)_2_D] and free (unbound to protein) 25(OH)D (47). However, total 25(OH)D measured in plasma or serum is an accepted indicator of vitamin D status, often used in epidemiological research, and it reflects both endogenous and dietary sources. It has a half-life of 2–3 weeks and is above all mostly used as a measure of vitamin D deficiency. However, the active form of vitamin D has an ability to modulate both innate and adaptive immunity, with pro-and anti-inflammatory actions that may be related to celiac disease (48).

The strengths of this study are the large study cohort, with study participants from six clinical sites in four countries, following the same study protocol to prospectively collect blood samples from birth up until 10 years of age before seroconversion to CDA.

This study adds additional knowledge regarding 25(OH)D status in genetically at-risk infants but raises the need for more studies and preferable randomized controlled trials to further investigate the role of 25(OH)D concentrations in early childhood and its relation to celiac disease onset.

In conclusion, this study shows that 25(OH)D concentrations <30 and >75 nmol/L during early infancy are associated with an increased risk of developing CDA in genetically at-risk children, indicating a possible role of 25(OH)D in the development of celiac disease.

## Data Availability Statement

The data presented in the study are deposited in the NIDDK Central Repository at https://repository.niddk.nih.gov/studies/teddy/.

## Ethics Statement

The study was conducted according to the guidelines of the Declaration of Helsinki, and local institutional or regional ethics review boards in all participating countries approved the study. Written informed consent to participate in this study was provided by the participants' legal guardian/next of kin.

## Author Contributions

CA: designed the study, drafted the initial manuscript, interpretation of data, and reviewed and revised the manuscript. DA: conceptualized and designed the study and critical review of both intermediary and final versions of the manuscript. WH, MR, J-XS, JT, A-GZ, BA, and JK: coordinated and supervised data collection, interpretation of data, and critical review of both intermediary and final versions of the manuscript. KK, SK, SV, and IE: interpretation of data and critical review of both intermediary and final versions of the manuscript. UU and MB: accusation of data collection, interpretation of data, and critical review of both intermediary and final versions of the manuscript. JN: conceptualized the study, interpretation of data, and critical review of both intermediary and final versions of the manuscript. XL: carried out the statistical analyses, interpretation of data, and reviewed and revised the manuscript. All authors approved the final manuscript as submitted and agree to be accountable for all aspects of the work, ensuring that questions related to the accuracy or integrity of any part of the work are appropriately investigated and resolved.

## TEDDY Study Group

Colorado Clinical Center: Marian Rewers, M.D., Ph.D., PI, Aaron Barbour, Kimberly Bautista, Judith Baxter, Daniel Felipe-Morales, Brigitte I. Frohnert, M.D., Marisa Stahl, M.D., Patricia Gesualdo, Rachel Haley, Michelle Hoffman, Rachel Karban, Edwin Liu, M.D., Alondra Munoz, Jill Norris, Ph.D., Stesha Peacock, Hanan Shorrosh, Andrea Steck, M.D., Megan Stern, Kathleen Waugh. University of Colorado, Anschutz Medical Campus, Barbara Davis Center for Childhood Diabetes.

Finland Clinical Center: Jorma Toppari, M.D., Ph.D., PI^¥∧^, Olli G. Simell, M.D., Ph.D., Annika Adamsson, Ph.D.^∧^, Sanna-Mari Aaltonen^∧^, Suvi Ahonen^*±§^, Mari Åkerlund^*±§^, Leena Hakola^*±^, Anne Hekkala, M.D.^μ^^¤^, Henna Holappa^μ^^¤^, Heikki Hyöty, M.D., Ph.D.^*±^, Anni Ikonen^μ^^¤^, Jorma Ilonen, M.D., Ph.D.^¥¶^, Sanna Jokipuu^∧^, Leena Karlsson^∧^, Jukka Kero M.D., Ph.D.^¥∧^, Miia Kähönen^μ^^¤^, Mikael Knip, M.D., Ph.D.^*±^, Minna-Liisa Koivikko^μ^^¤^, Katja Kokkonen^*±^, Merja Koskinen^*±^, Mirva Koreasalo^*±§^, Kalle Kurppa, M.D., Ph.D.^*±^, Salla Kuusela, M.D.^*±^, Jarita Kytölä^*±^, Sinikka Lahtinen^*±^, Jutta Laiho, Ph.D.^*^, Tiina Latva-aho^μ^^¤^, Laura Leppänen^∧^, Katri Lindfors, Ph.D.^*^, Maria Lönnrot, M.D., Ph.D.^*±^, Elina Mäntymäki^∧^, Markus Mattila^*±^, Maija Miettinen^§^, Katja Multasuo^μ^^¤^, Teija Mykkänen^μ^^¤^, Tiina Niininen^±*^, Sari Niinistö^§^, Mia Nyblom^*±^, Sami Oikarinen, Ph.D.^*±^, Paula Ollikainen^μ^^¤^, Zhian Othmani^¥^, Sirpa Pohjola ^μ^^¤^, Jenna Rautanen^±§^, Anne Riikonen^*±§^, Minna Romo^∧^, Satu Simell, M.D., Ph.D.^¥^, Aino Stenius^μ^^¤^, Päivi Tossavainen, M.D.^μ^^¤^, Mari Vähä-Mäkilä^¥^, Eeva Varjonen^∧^, Riitta Veijola, M.D., Ph.D.^μ^^¤^, Irene Viinikangas^μ^^¤^, Suvi M. Virtanen, M.D., Ph.D.^*±§^. ^¥^University of Turku, ^*^Tampere University, ^μ^University of Oulu, ^∧^Turku University Hospital, Hospital District of Southwest Finland, ^±^Tampere University Hospital,^¤^Oulu University Hospital, §Finnish Institute for Health and Welfare, Finland, ^¶^University of Kuopio.

Georgia/Florida Clinical Center: Jin-Xiong She, Ph.D., PI, Desmond Schatz, M.D.^*^, Diane Hopkins, Leigh Steed, Jennifer Bryant, Katherine Silvis, Michael Haller, M.D.^*^, Melissa Gardiner, Richard McIndoe, Ph.D., Ashok Sharma, Stephen W. Anderson, M.D.^∧^, Laura Jacobsen, M.D.^*^, John Marks, DHSc.^*^, P.D. Towe^*^. Center for Biotechnology and Genomic Medicine, Augusta University. ^*^University of Florida, Pediatric Endocrinology. ^∧^Pediatric Endocrine Associates, Atlanta.

Germany Clinical Center: Anette G. Ziegler, M.D., PI, Ezio Bonifacio Ph.D.^*^, Cigdem Gezginci, Anja Heublein, Eva Hohoff^¥^, Sandra Hummel, Ph.D., Annette Knopff, Charlotte Koch, Sibylle Koletzko, M.D.^¶^, Claudia Ramminger, Roswith Roth, Ph.D., Jennifer Schmidt, Marlon Scholz, Joanna Stock, Katharina Warncke, M.D., Lorena Wendel, Christiane Winkler, Ph.D. Forschergruppe Diabetes e.V. and Institute of Diabetes Research, Helmholtz Zentrum München, Forschergruppe Diabetes, and Klinikum rechts der Isar, Technische Universität München. ^*^Center for Regenerative Therapies, TU Dresden, ^¶^Dr. von Hauner Children's Hospital, Department of Gastroenterology, Ludwig Maximillians University Munich, ^¥^University of Bonn, Department of Nutritional Epidemiology.

Sweden Clinical Center: Åke Lernmark, Ph.D., PI, Daniel Agardh, M.D., Ph.D., Carin Andrén Aronsson, Ph.D., Maria Ask, Rasmus Bennet, Corrado Cilio, Ph.D., M.D., Susanne Dahlberg, Malin Goldman Tsubarah, Emelie Ericson-Hallström, Annika Björne Fors, Lina Fransson, Thomas Gard, Monika Hansen, Susanne Hyberg, Berglind Jonsdottir, M.D., Ph.D., Helena Elding Larsson, M.D., Ph.D., Marielle Lindström, Markus Lundgren, M.D., Ph.D., Marlena Maziarz, Ph.D., Maria Månsson Martinez, Jessica Melin, Zeliha Mestan, Caroline Nilsson, Yohanna Nordh, Kobra Rahmati, Anita Ramelius, Falastin Salami, Anette Sjöberg, Carina Törn, Ph.D., Ulrika Ulvenhag, Terese Wiktorsson, Åsa Wimar. Lund University.

Washington Clinical Center: William A. Hagopian, M.D., Ph.D., PI, Michael Killian, Claire Cowen Crouch, Jennifer Skidmore, Christian Chamberlain, Brelon Fairman, Arlene Meyer, Jocelyn Meyer, Denise Mulenga, Nole Powell, Jared Radtke, Shreya Roy, Davey Schmitt, Sarah Zink. Pacific Northwest Research Institute.

Pennsylvania Satellite Center: Dorothy Becker, M.D., Margaret Franciscus, MaryEllen Dalmagro-Elias Smith, Ashi Daftary, M.D., Mary Beth Klein, Chrystal Yates. Children's Hospital of Pittsburgh of UPMC.

Data Coordinating Center: Jeffrey P. Krischer, Ph.D., PI, Rajesh Adusumali, Sarah Austin-Gonzalez, Maryouri Avendano, Sandra Baethke, Brant Burkhardt, Ph.D., Martha Butterworth, Nicholas Cadigan, Joanna Clasen, Kevin Counts, Christopher Eberhard, Steven Fiske, Laura Gandolfo, Jennifer Garmeson, Veena Gowda, Belinda Hsiao, Christina Karges, Qian Li, Ph.D., Shu Liu, Xiang Liu, Ph.D., Kristian Lynch, Ph.D., Jamie Malloy, Cristina McCarthy, Jose Moreno, Hemang M. Parikh, Ph.D., Cassandra Remedios, Chris Shaffer, Susan Smith, Noah Sulman, Ph.D., Roy Tamura, Ph.D., Dena Tewey, Michael Toth, Ulla Uusitalo, Ph.D., Kendra Vehik, Ph.D., Ponni Vijayakandipan, Melissa Wroble, Jimin Yang, Ph.D., R.D., Kenneth Young, Ph.D. *Past staff: Michael Abbondondolo, Lori Ballard, Rasheedah Brown, David Cuthbertson, Stephen Dankyi, David Hadley, Ph.D., Kathleen Heyman, Francisco Perez Laras, Hye-Seung Lee, Ph.D., Colleen Maguire, Wendy McLeod, Aubrie Merrell, Steven Meulemans, Ryan Quigley, Laura Smith, Ph.D*. University of South Florida.

Project Scientist: Beena Akolkar, Ph.D. National Institutes of Diabetes and Digestive and Kidney Diseases.

Other Contributors: Thomas Briese, Ph.D., Columbia University. Todd Brusko, Ph.D., University of Florida. Suzanne Bennett Johnson, Ph.D., Florida State University. Eoin McKinney, Ph.D., University of Cambridge. Tomi Pastinen, M.D., Ph.D., The Children's Mercy Hospital. Eric Triplett, Ph.D., University of Florida.

Dietary Biomarkers Laboratory: Iris Erlund, Ph.D., Irma Salminen, Jouko Sundvall, Nina Kangas, Petra Arohonka. Finnish Institute for Health and Welfare, Helsinki, Finland.

## Conflict of Interest

The authors declare that the research was conducted in the absence of any commercial or financial relationships that could be construed as a potential conflict of interest.

## Publisher's Note

All claims expressed in this article are solely those of the authors and do not necessarily represent those of their affiliated organizations, or those of the publisher, the editors and the reviewers. Any product that may be evaluated in this article, or claim that may be made by its manufacturer, is not guaranteed or endorsed by the publisher.

## Abbreviations

25(OH)D: 25-hydroxyvitamin D
CDA: celiac disease autoimmunity
FDR: first degree relative
HLA: human leukocyte antigen
IA: islet autoimmunity
LDP: long distance protocol
SNP: single nucleotide polymorphisms
TEDDY: the environmental determinants of diabetes in the young
tTGA: tissue transglutaminase autoantibodies
T1D: type 1 diabetes.
