# Supplementary material for: 25(OH)D Levels in Infancy Is Associated With Celiac Disease Autoimmunity in At-Risk Children: A Case–Control Study
Source: Front Nutr. 2021 Aug 11;8:720041. doi: 10.3389/fnut.2021.720041 (PMC8479793; doi:10.3389/fnut.2021.720041)
Supplement: Supplementary file 1 [file Data_Sheet_1.docx]

**Supplemental data**

**Supplementary Table 1.** Plasma 25(OH)D concentrations by selected matching variables in the nested case control cohort.

|  |  | **Plasma 25(OH)D concentration (nmol/L)** | | | |
| --- | --- | --- | --- | --- | --- |
|  |  | **Early infancy** | | **Childhood** | |
| Matching variable | **Cases, n (%)** | **Cases** | **Controls** | **Cases** | **Controls** |
| **Clinical center** |  | Median (Q1, Q3) | Median (Q1, Q3) | Median (Q1, Q3) | Median (Q1, Q3) |
| Finland | 64 (22.7) | 37.0 (29.0, 49.0) | 41.5 (32.0, 50.0) | 42.6 (34.5, 49.4) | 43.2 (37.0, 52.6) |
| Germany | 27 (9.6) | 77.0 (51.0, 82.0) | 63.0 (40.0, 79.0) | 69.5 (58.0, 83.0) | 62.2 (50.2, 70.2) |
| Sweden | 116 (41.5) | 58.5 (42.0, 74.5) | 58.5 (47.0, 71.0) | 59.3 (49.2, 69.0) | 59.7 (49.0, 70.4) |
| Colorado | 36 (12.8) | 45.0 (27.0, 56.0) | 47.0 (26.0, 65.0) | 50.9 (43.7, 60.1) | 54.0 (43.3, 66.6) |
| Washington | 16 (5.7) | 31.5 (20.5, 46.0) | 50.0 (41.5, 67.0) | 48.2 (38.1, 52.7) | 61.0 (47.0, 73.3) |
| Georgia | 22 (7.8) | 54.0 (39.0, 71.0) | 52.0 (41.0, 58.0) | 52.5 (43.5, 71.5) | 55.5 (47.0, 66.0) |
| **Sex** |  |  |  |  |  |
| Female | 144 (48.6) | 49.0 (39.0, 73.0) | 50.5 (37.0, 64.0) | 52.3 (43.8, 67.3) | 55.0 (42.1, 67.0) |
| Male | 137 (51.4) | 48.5 (30.0, 65.0) | 52.0 (39.0, 66.0) | 54.5 (42.0, 66.4) | 56.0 (45.3, 65.3) |
| **FDR with T1D*** |  |  |  |  |  |
| Yes | 68 (24.2) | 51.5 (34.5, 80.0) | 54.0 (38.0, 66.0) | 57.3 (43.9, 76.0) | 57.0 (44.7, 65.4) |
| No | 213 (75.8) | 48.0 (35.0, 67.0) | 51.0 (38.0, 66.0) | 53.0 (43.0, 65.5) | 54.3 (43.3, 66.3) |

Footnote
* First degree relative (mother, father or sibling) with type 1 diabetes; FDR with type 1 diabetes (T1D)

**Supplementary Table 2.** Descriptive characteristics of subjects allocated in 25(OH)D concentrations categories; <30 nmol/L, 30-50 nmol/L, 50-75 nmol/L, and >75 nmol/L, at early infancy defined as the first available sample up to 12 months of age.

|  | **< 30 nmol/L**  (n=114) | **30-50 nmol/L**  (n = 284) | **50 – 75 nmol/L** (n= 307) | **>75 nmol/L**  (n = 130) |
| --- | --- | --- | --- | --- |
|  | **N (%)** | **N (%)** | **N (%)** | **N (%)** |
| Female sex (yes) | 54 (47.4) | 151 (53.2) | 147 (47.9) | 69 (53.1) |
| Clinical Center |  |  |  |  |
| Colorado | 31 (27.2) | 29 (10.2) | 42 (13.7) | 8 (6.2) |
| Georgia | 4 (3.5) | 20 (7.0) | 26 (8.5) | 7 (5.4) |
| Washington | 15 (13.2) | 16 (5.6) | 20 (6.5) | 5 (3.8) |
| Finland | 40 (35.1) | 115 (40.5) | 43 (14.0) | 8 (6.2) |
| Germany | 4 (3.5) | 15 (5.3) | 24 (7.8) | 25 (19.2) |
| Sweden | 20 (17.5) | 89 (31.3) | 152 (49.5) | 77 (59.2) |
| HLA-genotype |  |  |  |  |
| DR3/3 | 27 (23.7) | 65 (22.9) | 67 (21.8) | 27 (20.8) |
| DR3/X | 44 (38.6) | 111 (39.1) | 125 (40.7) | 56 (43.1) |
| Other | 34 (48.6) | 88 (47.6) | 104 (45.2) | 32 (42.7) |
| Persistent confirmed islet autoantibodies (yes)# | 36 (31.6) | 87 (30.6) | 66 (21.5) | 28 (21.5) |
| FDR with type 1 diabetes (yes) | 24 (21.1) | 60 (21.1) | 78 (25.4) | 36 (27.7) |
| FDR with celiac disease (yes) | 7 (6.1) | 21 (7.4) | 10 (3.3) | 8 (6.2) |
| Season of birth |  |  |  |  |
| Spring (Mar – May) | 23 (20.2) | 81 (28.5) | 72 (23.5) | 25 (19.2) |
| Summer (Jun – Aug) | 19 (16.7) | 72 (25.4) | 82 (26.7) | 41 (31.5) |
| Fall (Sep – Nov) | 44 (38.6) | 51 (18.0) | 81 (26.4) | 31 (23.8) |
| Winter (Dec – Feb) | 28 (24.6) | 80 (28.2) | 72 (23.5) | 33 (25.4) |
| Season of blood draw |  |  |  |  |
| Spring (Mar – May) | 35 (30.7) | 76 (26.8) | 88 (28.7) | 42 (32.3) |
| Summer (Jun – Aug) | 19 (16.7) | 81 (28.5) | 78 (25.4) | 26 (20.0) |
| Fall (Sep – Nov) | 21 (18.4) | 77 (27.1) | 85 (27.7) | 40 (30.8) |
| Winter (Dec – Feb) | 39 (34.2) | 50 (17.6) | 56 (18.2) | 22 (16.9) |
| Maternal education |  |  |  |  |
| Higher education (yes) | 96 (84.2) | 235 (83.3) | 214 (69.9) | 100 (76.9) |
| Maternal vitamin D supplementation during pregnancy (yes) | 79 (69.3) | 188 (66.2) | 199 (64.8) | 83 (63.8) |
| Exclusive breastfeeding status (yes)* | 39 (34.2) | 47 (16.5) | 30 (9.8) | 19 (14.6) |
| Breastfeeding status (yes)* | 111 (97.4) | 216 (76.1) | 151 (49.2) | 88 (67.7) |
| Introduced to gluten (yes) * | 20 (17.5) | 75 (26.4) | 130 (42.3) | 50 (38.5) |
| Vitamin D supplement use (yes) * | 63 (55.3) | 211 (74.3) | 224 (73.0) | 119 (91.5) |
| Long-distance protocol (yes)* | 6 (5.3) | 13 (4.6) | 29 (9.4) | 18 (13.8) |
| Age at 1^st^ available sample (months), median (IQR) | 3.6 (3.2, 4.0) | 3.8 (3.4, 6.0) | 4.2 (3.6, 6.8) | 4.4 (3.7, 6.4) |

# Persistent islet autoantibody positive at time of the nested case-control study design.
* At the first available sample at visits up to 12 months of age

**Supplemental data**

Children enrolled in TEDDY
 n = 8676

IA 1:3 NCC matched sets
n = 418

T1D 1:3 NCC matched sets
n = 114

CDA case-control matched sets
n = 287

Matched sets without 25(OH)D concentrations data on cases or controls
n = 6

CDA case-control matched sets with 25(OH)D concentrations
 n = 281

**Legend for Supplementary Figure 1.**
Flow chart describing the selection of the CDA case–control sets (1:3) for the study.
CDA, celiac disease autoimmunity; IA; islet autoantibody; NCC, nested case–control; T1D, type 1 diabetes. Cases and controls were matched on clinical center, sex and FDR with T1D
